# *Mycobacterium microti* Infections in Free-Ranging Red Deer (*Cervus elaphus*)

**DOI:** 10.3201/eid2708.210634

**Published:** 2021-08

**Authors:** Giovanni Ghielmetti, Anne M. Kupca, Matthias Hanczaruk, Ute Friedel, Hubert Weinberger, Sandra Revilla-Fernández, Erwin Hofer, Julia M. Riehm, Roger Stephan, Walter Glawischnig

**Affiliations:** Institute for Food Safety and Hygiene, Section of Veterinary Bacteriology, Vetsuisse Faculty University of Zurich, Zurich, Switzerland (G. Ghielmetti, U. Friedel, R. Stephan);; Bavarian Health and Food Safety Authority, Oberschleissheim, Germany (A.M. Kupca, M. Hanczaruk, J.M. Riehm);; Institute for Veterinary Disease Control, Austrian Agency for Health and Food Safety (AGES), Innsbruck and Mödling, Austria (H. Weinberger, S. Revilla-Fernández, E. Hofer, W. Glawischnig)

**Keywords:** animal hosts, Austria, bacteria, Cervus elaphus, disease reservoirs, epidemics, epidemiology, Germany, MLVA, multilocus variable-number tandem-repeat analysis, Mycobacterium microti, Mycobacterium tuberculosis complex, outbreaks, red deer, respiratory infections, tuberculosis and other mycobacteria, whole-genome sequencing, zoonoses

## Abstract

Infections with *Mycobacterium microti*, a member of the *M. tuberculosis* complex, have been increasingly reported in humans and in domestic and free-ranging wild animals. At postmortem examination, infected animals may display histopathologic lesions indistinguishable from those caused by *M. bovis* or *M. caprae*, potentially leading to misidentification of bovine tuberculosis. We report 3 cases of *M. microti* infections in free-ranging red deer (*Cervus elaphus*) from western Austria and southern Germany. One diseased animal displayed severe pyogranulomatous pleuropneumonia and multifocal granulomas on the surface of the pericardium. Two other animals showed alterations of the lungs and associated lymph nodes compatible with parasitic infestation. Results of the phylogenetic analysis including multiple animal strains from the study area showed independent infection events, but no host-adapted genotype. Personnel involved in bovine tuberculosis–monitoring programs should be aware of the fastidious nature of *M. microti*, its pathogenicity in wildlife, and zoonotic potential.

Tuberculosis (TB) is one of the most prevalent zoonotic diseases worldwide and remains the leading cause of death from a single infectious agent (*1*). The causative pathogens of TB in humans and animals are a group of closely related acid-fast bacilli commonly known as the *Mycobacterium tuberculosis* complex (MTBC). One animal-adapted sublineage within the complex, *M. microti*, was first isolated from field voles (*Microtus agrestis*) that had granulomatous tuberculosis-like lesions (*2*). Although wild rodents, such as bank voles (*Myodes glareolus*), wood mice (*Apodemus sylvaticus*), and shrews (*Sorex araneus*), are considered to be primary reservoirs for *M. microti*, several other hosts have been identified, including domestic and wild animals (*3*,*4*). Overall, cats (*5*,*6*), New World camelids (*7*), and free-ranging wild boar (*8*–*10*) seem to be prone to *M. microti* infections; humans (*11*–*14*) and other animal species, including pigs (*15*), goats (*16*), cattle (*17*,*18*), dogs (*19*), captive meerkats (*20*), squirrel monkeys (*21*), and ferrets (*14*), are most likely incidental hosts.

This broad host range, however, highlights the pathogenic potential of *M. microti* and the need to reveal its virulence mechanisms. Comparative genomics studies have identified >100 genes whose presence are facultative and differ among members of MTBC. Many of these genes occur in chromosomal regions of difference (RD) that have been deleted from certain species and that may confer differences in phenotype, host range, and virulence (*22*). Isolates of the animal-adapted ecotype defined as *M. microti* are characterized by the deletion of the RD1^mic^ in the RD1 region, which includes open reading frame coding for well-known virulence factors, such as early secreted antigenic target (ESAT) 6, locus tag Rv3875, and CFP-10, a culture filtrate protein encoded by the neighboring gene Rv3874 (*23*). Strains lacking RD1 are likely to be less virulent or pathogenic than other members of the MTBC possessing an intact locus (*22*). However, pulmonary and disseminated *M. microti* infections have been described in both immunocompromised and immunocompetent human patients in different countries in Europe (*11*,*12*,*14*,*24*). Until recently, reports of *M. microti* infections were geographically restricted to continental Europe and the United Kingdom. However, a recent study from South Africa revealed the presence of this *Mycobacterium* species in 1.9% of local human tuberculosis cases (*25*). These findings highlight the potential of *M. microti* to cause clinical illness in immunocompetent patients and suggest that the pathogenicity of certain strains is higher than previously estimated. Therefore, it is crucial to identify clinical MTBC isolates at the species level, and the zoonotic risk posed by *M. microti* should be further evaluated.

The mode of infection of *M. microti* can only be speculated for humans, animals, and in particular herbivores, such as free-ranging red deer. Similar to that for *M. caprae*, transmission of *M. microti* is likely to occur indirectly through a contaminated environment. Wounds in the oral cavity may play an important role as entry ports for *M. microti*; involvement of the lungs, heart, and eventually additional organs is most likely a consequence of bacteremia, as it is in other animal species (*3*,*5*). The first confirmed *M. caprae* TB case in deer in western Austria was recorded in 1998. Subsequent infections in cattle, deer, and humans were reported in the same area (*26*). As a consequence, an ongoing wildlife surveillance program monitoring *M. caprae* in deer was started in 2008 (*27*). Furthermore, Germany in 2007 and Switzerland in 2013 reported anecdotal outbreaks in cattle (*28*,*29*). During 2011–2013, a monitoring program coordinated by the EMIDA ERA-Net (Coordination of European Research on Emerging and Major Infectious Diseases of Livestock European Research Area Networks) partnership and including specific regions of Austria, Switzerland, Germany, and Italy was conducted with the aim of investigating the prevalence of bovine tuberculosis (bTB) in red deer and additional wildlife species such as wild boar, chamois, and roe deer (*30*,*31*). We report 3 TB cases in red deer identified within the framework of these monitoring programs.

## Study Material and Methods

### Cases

Three cases of natural *M. microti* infections in red deer were identified (Table 1). The deer in case 1, a highly emaciated 9-year-old stag from the province of Vorarlberg, Austria, was humanely killed by a local game warden, who submitted the lungs, heart, and lymphatic tissues (including medial retropharyngeal, tracheobronchial, and mediastinal lymph nodes) fresh for pathoanatomic inspection. Thereafter, histologic examination and mycobacterial analysis of the lungs were performed. The deer in case 2 was a stag 1–3 years of age and in case 3 a hind >2 years of age, both in the province of Miesbach, Germany, where deer are regularly hunted. The heads, lungs, intestines, and associated lymph nodes were macroscopically inspected; subsequently, histopathologic and bacteriologic examinations of the lungs and lymph nodes were performed.

**Table 1:** Overview of 3 cases of tuberculosis caused by *Mycobacterium microti* in red deer, Austria and Germany

Case	Age, y/sex	Year isolated	Main findings	Country
1	9/M	2017	Severe pyogranulomatous pleuropneumonia, multifocal to coalescing granulomas on the epicardium	Austria
2	1–3/M	2013	Moderate focal nonpurulent pneumonia	Germany
3	>2/F	2013	Moderate purulent bronchitis and bronchiolitis, fibroblastic pleuritis, lungworms	Germany

### Mycobacterial Analyses and Histologic Examination 

We isolated mycobacteria following a standardized protocol as described elsewhere (*32*). In brief, 2–3 g of minced tissue samples were homogenized in 5 mL 0.9% NaCl solution by using a rotating-blade macerator system (Ultra-Turrax IKA, https://www.ika.com). The suspension was decontaminated by using 1% N-acetyl-L-cystein-NaOH solution and neutralized with 20 mL phosphate buffer (pH 6.8). We centrifuged the solution for 20 min at 3300 × *g* and plated the obtained pellet on 2 growth media: Löwenstein-Jensen medium with glycerin and PACT (polymyxin B, amphotericin B, carbenicillin, and trimethoprim) and Stonebrink medium with pyruvate and PACT (BD, https://www.bd.com). Cultures of lung and lymph node specimens on solid Stonebrink medium yielded growth of suspicious mycobacterial colonies after 4–6 wk of incubation at 37°C. The isolates were identified by using GenoType MTBC reversed line blotting (Hain Lifescience, https://www.hain-lifescience.de). For histologic examination, we fixed tissue samples in 10% nonbuffered formalin for ≈48 h, then trimmed and routinely embedded them in paraffin wax. Sections of 3–4 μm were prepared and stained with hematoxylin and eosin (HE) and Ziehl Neelsen (ZN) or modified ZN (*33*).

### Investigation of Phylogenetic Relationships 

DVR spoligotyping (direct variable repeat spacer oligonucleotide typing) was performed using a commercial microarray system (Alere Technologies, https://www.globalpointofcare.abbott) with integrated data analysis as described elsewhere (*29*). Multilocus variable-number tandem repeats analysis (MLVA) was conducted based on the 24-loci panel standardized for *M. tuberculosis* typing (*34*). We amplified the single markers by endpoint PCR and subsequently analyzed them by using a capillary electrophoresis device (*29*). To investigate the phylogenetic relationships between the 3 isolates from red deer, we analyzed 8 additional strains isolated from different wild and domestic hosts that originated from the regions bordering Germany, Austria, and Switzerland by MLVA (Table 2). We calculated a neighbor-joining phylogenic tree based on the copy numbers of the tested loci using the MIRU-VNTR*plus* (https://www.miru-vntrplus.org/MIRU) server and exported it using MEGAX version 10.11 (*35*).

**Table 2:** Multilocus variable-number tandem-repeat analysis of 8 *Mycobacterium microti* strains used in study of tuberculosis caused by *M. microti* in red deer, Austria and Germany

Strain	Year isolated	Host	Country	Reference
TG 481	2010	Wild boar	Switzerland	(*31*)
TG 435	2010	Wild boar	Switzerland	(*31*)
TI 17–1545	2017	Wild boar	Switzerland	(*9*)
TG 15–1955	2015	Cat	Switzerland	(*5*)
TG 15–294	2015	Cat	Switzerland	This study
ZH 1522744	2016	Cat	Switzerland	(*5*)
18–2304	2016	Red fox	Austria	This study
SG 17–2287	2017	Alpaca	Switzerland	This study

## Results

### Case 1

**Figure 1:**
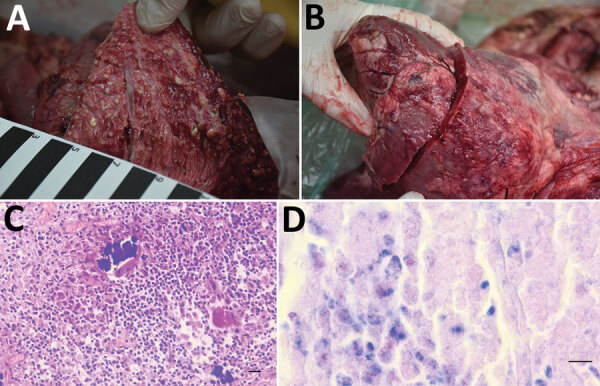
Macroscopic and histopathologic features in the red deer in case 1 in study of tuberculosis caused by *Mycobacterium microti* in red deer, Austria and Germany. A) Gross picture of the cutting surface of the lungs with severe pyogranulomatous pleuropneumonia with multifocal to confluent cavernous granulomas, 2–10 mm diameter. B) Multifocal to coalescing granulomas 4–25 mm diameter on the surface of the epicardium. C) Chronic multifocal to coalescing pyogranulomatous pneumonia in lungs with central areas of necrosis and mineralization surrounded by numerous epithelioid macrophages and a few multinucleated Langhans giant cells. Single lymphocytes and plasma cells were observed around the periphery and between the granulomas, hematoxylin and eosin stain. Scale bar = 20 μm. D) Numerous macrophages and epithelioid cells containing solitary or multiple acid-fast bacilli. Ziehl Neelsen stain. Scale bar = 10 μm.

Postmortem examination of the stag revealed multiple enlarged lymph nodes exhibiting a whitish cut surface. The lung tissue showed severe pyogranulomatous pleuropneumonia with multifocal to confluent cavernous granulomas of 2–10 mm diameter (Figure 1, panel A). Multifocal to coalescing granulomas of 4–25 mm diameter were observed on the surface of the epicardium (Figure 1, panel B). Histopathologic examination of the lung revealed a severe chronic multifocal to coalescing pyogranulomatous pneumonia with focal areas of fibrosis, central areas of necrosis and mineralization, surrounded by numerous epithelioid macrophages and a few multinucleated Langhans giant cells. Lymphocytes, plasma cells and occasionally well-differentiated fibroblasts surrounded the granulomas (Figure 1, panel C). Few extracellular and intracellular acid-fast bacilli were identified in the pulmonary lesions by using ZN staining (Figure 1, panel D).

### Case 2

**Figure 2:**
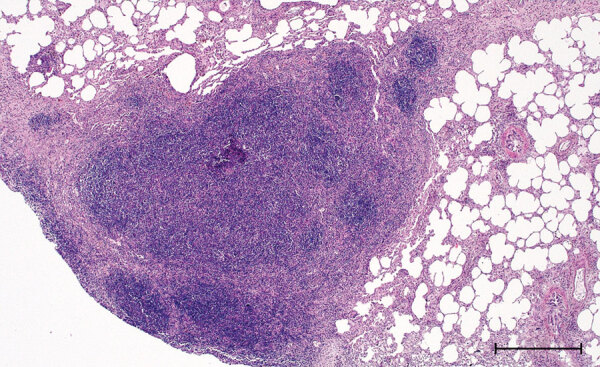
Histopathologic features in red deer in case 2 in study of tuberculosis caused by *Mycobacterium microti* in red deer, Austria and Germany. Lung tissue highly infiltrated by round cells, predominantly lymphocytes and some macrophages, single multinucleated Langhans-type giant cells, hematoxylin and eosin stain. Scale bar = 500 μm.

Macroscopically, a single yellowish, pinhead-sized focus in the left dorsal main lobe of the lung of this stag was observed. Lymph nodes and intestines did not display any abnormalities. Histologically, the pulmonary focus consisted of macrophages and lymphocytes with single multinucleated Langhans-type giant cells in the lesion, surrounded by eosinophilic lymphocytes (Figure 2). Numerous eosinophilic granulocytes were seen in the pulmonary lymph node. These findings were compatible with a parasitic infestation. Intracellular acid-fast bacilli could not be identified by using modified ZN staining. 

### Case 3

**Figure 3:**
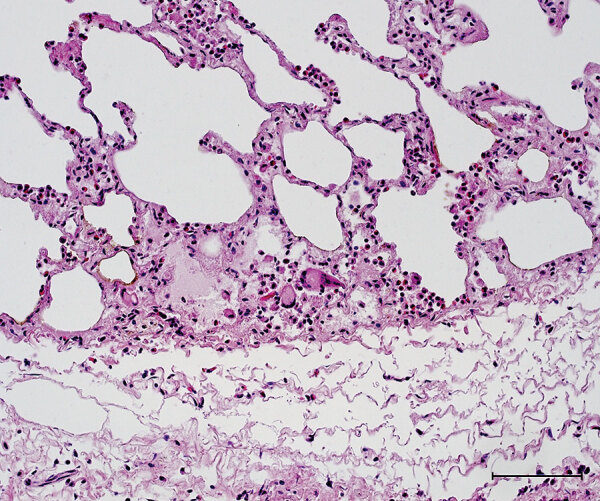
Histopathologic features in red deer in case 3 in study of tuberculosis caused by *Mycobacterium microti* in red deer, Austria and Germany. Lung tissue with granulocytic infiltration and some multinucleated Langhans-type giant cells, hematoxylin and eosin stain. Scale bar = 100 μm.

The caudal part of the main lobes of the lung of this hind showed multiple whitish foci <0.5 cm in size. Enlarged pulmonary lymph nodes and multifocal fibroblastic pleuritis were observed. The histologic examination revealed moderate purulent bronchitis and bronchiolitis with several intraluminal stages of lungworms and infiltration of numerous eosinophilic granulocytes. We observed very few multinucleated Langhans-type giant cells (Figure 3) and could identify no intracellular acid-fast bacilli in the lesions using modified ZN staining.

### Investigation of Phylogenetic Relationships

**Figure 4:**
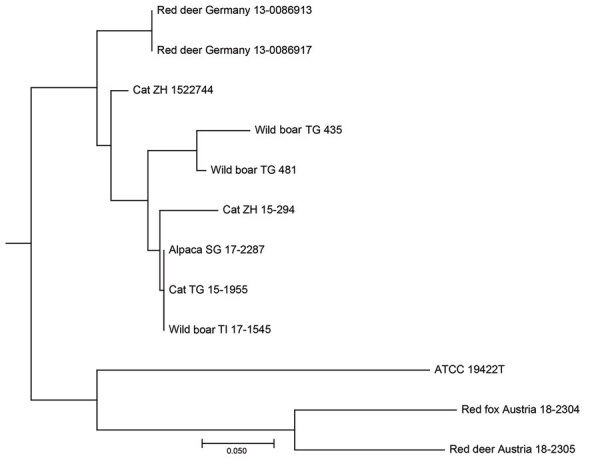
Neighbor-joining tree based on the copy numbers of 24-loci mycobacterial interspersed repetitive unit variable-number tandem-repeat analysis derived from 11 *Mycobacterium microti* clinical isolates and type strain *M. microti* Reed ATCC 19422^T^ in study of tuberculosis caused by *M. microti* in red deer, Austria and Germany. We calculated the tree using the MIRU-VNTR*plus* server (https://www.miru-vntrplus.org; Appendix) and exported it using MEGAX version 10.11 (https://www.megasoftware.net). Scale bar indicates substitutions per site.

**Figure 5:**
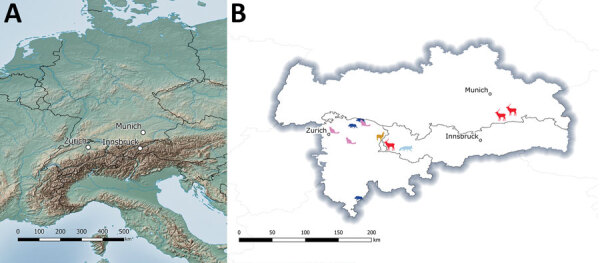
Geographic distribution of tuberculosis cases caused by *Mycobacterium microti* in different animal species over 8 years from study of tuberculosis caused by *M. microti* in red deer, Austria and Germany. Central Europe (left) and the region bordering Germany, Austria, and Switzerland (right) are shown. Animals are shaped and colored: red deer, red; cats, pink; wild boar, dark blue; alpaca, orange; and red fox, light blue.

The isolates from 11 animals (3 wild boars, 3 cats, 1 alpaca, and 1 red fox), integrated for further comparative genotyping, exhibited the same spoligotype signature, SB0118, characterized by the presence of spacers 37–38 (https://www.mbovis.org). The same signature is also registered in the international spoligotyping database SpolDB4 as ST 539 and is characteristic of *M. microti* (*36*). MLVA showed 2 distinct genotypes (Figure 4; Appendix), 1 for the 2 identical isolates from the red deer from Germany and 1 for the red deer isolate from Austria. Of interest, the isolates from Germany were closely related to isolates from Switzerland, whereas 2 isolates from Austria, originating from a red deer (case 1) and a red fox, were genetically more distant despite their geographic proximity (Figure 5). 

## Discussion

Case 1 in this study reported an *M. microti*–positive stag killed in the alpine region in western Austria manifesting clinical signs of tuberculosis. Tuberculosis caused by *M. caprae* has been described several times in domestic animals and wildlife in this area (*26*,*30*). Specifically, red deer represent a reservoir and a possible source of infection with *M. caprae* for cattle in Austria, Germany, Italy, and Switzerland (*26*,*28*,*29*,*37*). *M. microti* has been isolated only once in Austria, from a red fox without visible lesions (Table 2). This fox was located at a distance of ≈30 km from the site where the *M. microti*–positive stag in case 1 was found. Clear evidence proving transmission of *M. microti* between individual animals of the same species or between species is missing. Human-to-human transmission regarding this pathogen has previously been investigated and the possibility cannot be dismissed (*14*). However, ingesting feed or water from contaminated sources, for example, might play an important role in transmitting mycobacteria to wildlife. In fact, recent reports suggest that *M. microti* infections might often occur through oral ingestion and that direct transmission between animals is less likely (*9*,*20*).

The presence of MTBC in wild red deer seems to depend on multiple factors, such as population density, TB prevalence in nearby cattle or other wildlife species, and the morphologic structure of the habitat. Observations made from infected wild deer in New Zealand showed that *M. bovis* prevalence decreased substantially after control of TB-infected possums, suggesting that wild deer may be spillover hosts that can be regularly reinfected by possums (*38*). In fact, considering the high levels (>50%) of bTB in cattle in Europe before eradication campaigns, sporadic transmissions to wildlife populations might have occurred. It is, however, surprising to note that as a result of successfully lowering the prevalence in cattle, the disease has been eradicated from wild deer populations, such as in Switzerland (*38*). The situation is considerably different for deer in captivity or in high-density populations, which could be the origin of TB dissemination to other species (*39*). Of interest, the virulence of *M. microti* seems to vary greatly both between host species and within the same species. Most visible lesions compatible with TB diagnosed in *M. bovis*– and *M. caprae*–infected red deer are located in the lymph nodes, particularly the medial retropharyngeal and mesenteric lymph nodes, suggesting oral rather than aerosol transmission. The respiratory tract, including the lungs and associated lymph nodes, seems to be affected by MTBC in a secondary phase of the infection, which is also likely for *M. microti* infections in red deer (*30*).

DVR spoligotyping analysis is a popular technique worldwide for molecular characterization of MTBC of animal origin with excellent resolution and cost-benefit ratio (*40*,*41*), but the discriminatory power is too low to prove any link on an epidemiologic level or even minute transmission patterns among *M. microti* lineages. The molecular background of *M. microti* seems highly conserved, and traceback analyses are delicate. The 11 isolates included in this study were collected over an 8-year period from regions bordering Austria, Germany, and Switzerland (Table 2). These strains originated from 5 different wild and domestic animal species, and most of the animals in these cases showed severe TB lesions. Although the number of isolates investigated was small, in accordance with previous studies, no correlation between host species and *M. microti* genotypes was observed (*4*,*42*). Moreover, even though the isolates from Switzerland were genetically close, genetic variation determined by MLVA did not correlate with the relative geographic distance of their origin. In fact, the isolates from red deer in Germany were genetically closer to the strains from Switzerland, whereas the 2 isolates from Austria, originating in the border region shared with Switzerland, were genetically more distant (Figures 4, 5), which suggests that the circulation of host-adapted *M. microti* genetic lineages is unlikely. MLVA has been successfully used worldwide as an ancillary tool for animal TB epidemiologic surveillance and outbreak investigations in multihost scenarios (*27*,*43*–*45*). However, the discriminatory capacity of whole-genome sequencing has elsewhere been shown to be superior for identifying MTBC strains belonging to the same regional clonal complex, which may apply to *M. microti* as well (*39*,*46*). Under certain specific circumstances, such as for formalin-fixed, paraffin-embedded samples or extremely fastidious strains, MLVA represents a valid alternative to whole-genome sequencing. 

These findings highlight the wide host range of *M. microti* and suggest that it might be an opportunistic pathogen rather than a host-adapted MTBC member, such as *M. tuberculosis*. In the past, similar to *Mycobacterium bovis* BCG strains, certain vole strains of *M. microti* have been used to develop live attenuated human TB vaccines in the United Kingdom and the former Czechoslovakia (*47*–*49*). Therefore, marked virulence differences between *M. microti* strains are likely to exist (*50*).

Some seemingly feasible theories about the natural transmission route of *M. microti* suggest that the natural foci and reservoirs of this animal-adapted lineage are small rodents and that the pathogen subsequently infects predators, such as cats or foxes, through ingestion; the mode of infection for herbivores, such as red deer or alpacas, remains ill defined. The lesions we observed in the lungs of the deer in case 1, however, provide strong evidence of bacterial shedding, which might occur either as a consequence of inflammatory processes that break into the airways or by infection of alveolar macrophages that are part of the exudate (*5*), which result in environmental contamination and further transmission of the pathogen (*3*). It is therefore alarming that animal species, such as red deer, that can cover long distances in short periods of time might contribute to the spread of *M. microti*, an MTBC agent.

Because of the potential zoonotic risk related to MTBC members, rapidly and accurately identifying the mycobacterial species causing disease in animals hunted for human consumption is crucial. Once MTBC is detected, determining whether *M. bovis* or *M. caprae* is present is of primary importance for veterinary and public health authorities. To date, molecular testing of cultured bacteria remains the preferred method for differentiating MTBC species. Because of the fastidious nature of *M. microti* and the extremely slow growth rate of specific animal strains, this differentiation can take several months or remain incomplete in cases where the mycobacterium cannot be cultured. On the basis of published data, it can be assumed that a large proportion of *M. microti* infections remain culture negative, even if the incubation time is prolonged to 18 weeks (*8*). Therefore, identifying species using molecular methods on native samples is recommended.

These findings show the morphologic versatility of lesions induced by *M. microti* in red deer. Given the absence of typical pulmonary lesions in some cases, such as in the red deer in cases 2 and 3, diagnostic pathologists must remain highly alert. Incidence of this pathogen should be monitored within the framework of bTB surveillance programs and suspicious cases differentiated from classical bTB caused by *M. bovis* and *M. caprae*. The actual occurrence of *M. microti* in wildlife may be underestimated, and personnel involved in bTB monitoring programs should be aware of its pathogenicity and zoonotic potential. Therefore, molecular methods to differentiate this member of the MTBC should be included in the diagnostic workflow of bTB reference laboratories.

Appendix Additional information on strains of *Mycobacterium microti* identified in Germany, Austria, and Switzerland.

## References

[1] World Health Organization. Global tuberculosis report 2020. Geneva: World Health Organization. 2020 [cited 2021 Mar 17]. https://www.who.int/publications/i/item/9789240013131

[2] Wells AQ, Robb-Smith AHT. The murine type of tubercle bacillus (the vole acid-fast bacillus); with notes on the morphology of infection by the vole acid-fast bacillus. London: H.M. Stationery Office; 1946.

[3] Kipar A, Burthe SJ, Hetzel U, Rokia MA, Telfer S, Lambin X, et al. *Mycobacterium microti* tuberculosis in its maintenance host, the field vole (*Microtus agrestis*): characterization of the disease and possible routes of transmission. Vet Pathol. 2014;51:903–14. 10.1177/0300985813513040 24334995 PMC4225454

[4] Smith NH, Crawshaw T, Parry J, Birtles RJ. *Mycobacterium microti*: More diverse than previously thought. J Clin Microbiol. 2009;47:2551–9. 10.1128/JCM.00638-09 19535520 PMC2725668

[5] Peterhans S, Landolt P, Friedel U, Oberhänsli F, Dennler M, Willi B, et al. *Mycobacterium microti*: not just a coincidental pathogen for cats. Front Vet Sci. 2020;7:590037. 10.3389/fvets.2020.590037 33344530 PMC7744565

[6] Rüfenacht S, Bögli-Stuber K, Bodmer T, Jaunin VF, Jmaa DC, Gunn-Moore DA. *Mycobacterium microti* infection in the cat: a case report, literature review and recent clinical experience. J Feline Med Surg. 2011;13:195–204. 10.1016/j.jfms.2011.01.012 21338944 PMC11148936

[7] Oevermann A, Pfyffer GE, Zanolari P, Meylan M, Robert N. Generalized tuberculosis in llamas (*Lama glama*) due to *Mycobacterium microti.* J Clin Microbiol. 2004;42:1818–21. 10.1128/JCM.42.4.1818-1821.2004 15071059 PMC387549

[8] Boniotti MB, Gaffuri A, Gelmetti D, Tagliabue S, Chiari M, Mangeli A, et al. Detection and molecular characterization of *Mycobacterium microti* isolates in wild boar from northern Italy. J Clin Microbiol. 2014;52:2834–43. 10.1128/JCM.00440-14 24871212 PMC4136184

[9] Ghielmetti G, Hilbe M, Friedel U, Menegatti C, Bacciarini L, Stephan R, et al. Mycobacterial infections in wild boars (*Sus scrofa*) from Southern Switzerland: Diagnostic improvements, epidemiological situation and zoonotic potential. Transbound Emerg Dis. 2021;68:573–86. 10.1111/tbed.13717 32640107 PMC8247353

[10] Pérez de Val B, Sanz A, Soler M, Allepuz A, Michelet L, Boschiroli ML, et al. *Mycobacterium microti* infection in free-ranging wild boar, Spain, 2017–2019. Emerg Infect Dis. 2019;25:2152–4. 10.3201/eid2511.190746 31625855 PMC6810215

[11] Niemann S, Richter E, Dalügge-Tamm H, Schlesinger H, Graupner D, Königstein B, et al. Two cases of *Mycobacterium microti* derived tuberculosis in HIV-negative immunocompetent patients. Emerg Infect Dis. 2000;6:539–42. 10.3201/eid0605.000516 10998387 PMC2627952

[12] Panteix G, Gutierrez MC, Boschiroli ML, Rouviere M, Plaidy A, Pressac D, et al. Pulmonary tuberculosis due to *Mycobacterium microti*: a study of six recent cases in France. J Med Microbiol. 2010;59:984–9. 10.1099/jmm.0.019372-0 20488936

[13] van de Weg CAM, de Steenwinkel JEM, Miedema JR, Bakker M, van Ingen J, Hoefsloot W. The tough process of unmasking the slow-growing mycobacterium: case report of *Mycobacterium microti* infection. Access Microbiol. 2019;2:acmi000074. 33062933 10.1099/acmi.0.000074 PMC7525059

[14] van Soolingen D, van der Zanden AGM, de Haas PEW, Noordhoek GT, Kiers A, Foudraine NA, et al. Diagnosis of *Mycobacterium microti* infections among humans by using novel genetic markers. J Clin Microbiol. 1998;36:1840–5. 10.1128/JCM.36.7.1840-1845.1998 9650922 PMC104938

[15] Taylor C, Jahans K, Palmer S, Okker M, Brown J, Steer K. *Mycobacterium microti* isolated from two pigs. Vet Rec. 2006;159:59–60. 10.1136/vr.159.2.59-a 16829603

[16] Michelet L, de Cruz K, Phalente Y, Karoui C, Hénault S, Beral M, et al. *Mycobacterium microti* infection in dairy goats, France. Emerg Infect Dis. 2016;22:569–70. 10.3201/eid2203.151870 26890061 PMC4766897

[17] Jahans K, Palmer S, Inwald J, Brown J, Abayakoon S. Isolation of *Mycobacterium microti* from a male Charolais-Hereford cross. Vet Rec. 2004;155:373–4. 15493609

[18] Michelet L, de Cruz K, Tambosco J, Hénault S, Boschiroli ML. *Mycobacterium microti* interferes with bovine tuberculosis surveillance. Microorganisms. 2020;8:1850. 10.3390/microorganisms8121850 33255311 PMC7761213

[19] Deforges L, Boulouis HJ, Thibaud JL, Boulouha L, Sougakoff W, Blot S, et al. First isolation of *Mycobacterium microti* (Llama-type) from a dog. Vet Microbiol. 2004;103:249–53. 10.1016/j.vetmic.2004.06.016 15504596

[20] Palgrave CJ, Benato L, Eatwell K, Laurenson IF, Smith NH. *Mycobacterium microti* infection in two meerkats (*Suricata suricatta*). J Comp Pathol. 2012;146:278–82. 10.1016/j.jcpa.2011.06.001 21783200

[21] Henrich M, Moser I, Weiss A, Reinacher M. Multiple granulomas in three squirrel monkeys (*Saimiri sciureus*) caused by *Mycobacterium microti.* J Comp Pathol. 2007;137:245–8. 10.1016/j.jcpa.2007.06.005 17888448

[22] Pym AS, Brodin P, Brosch R, Huerre M, Cole ST. Loss of RD1 contributed to the attenuation of the live tuberculosis vaccines *Mycobacterium bovis* BCG and *Mycobacterium microti.* Mol Microbiol. 2002;46:709–17. 10.1046/j.1365-2958.2002.03237.x 12410828

[23] Berthet FX, Rasmussen PB, Rosenkrands I, Andersen P, Gicquel B. A *Mycobacterium tuberculosis* operon encoding ESAT-6 and a novel low-molecular-mass culture filtrate protein (CFP-10). Microbiology (Reading). 1998;144:3195–203. 10.1099/00221287-144-11-3195 9846755

[24] Xavier Emmanuel F, Seagar A-L, Doig C, Rayner A, Claxton P, Laurenson I. Human and animal infections with *Mycobacterium microti*, Scotland. Emerg Infect Dis. 2007;13:1924–7. 10.3201/eid1312.061536 18258049 PMC2876740

[25] Maguga-Phasha NTC, Munyai NS, Mashinya F, Makgatho ME, Mbajiorgu EF. Genetic diversity and distribution of *Mycobacterium tuberculosis* genotypes in Limpopo, South Africa. BMC Infect Dis. 2017;17:764. 10.1186/s12879-017-2881-z 29233106 PMC5727936

[26] Prodinger WM, Eigentler A, Allerberger F, Schönbauer M, Glawischnig W. Infection of red deer, cattle, and humans with *Mycobacterium bovis* subsp. *caprae* in western Austria. J Clin Microbiol. 2002;40:2270–2. 10.1128/JCM.40.6.2270-2272.2002 12037107 PMC130709

[27] Schoepf K, Prodinger WM, Glawischnig W, Hofer E, Revilla-Fernandez S, Hofrichter J, et al. A two-years’ survey on the prevalence of tuberculosis caused by *Mycobacterium caprae* in red deer (*Cervus elaphus*) in the Tyrol, Austria. ISRN Vet Sci. 2012;2012:245138. 10.5402/2012/245138 23762580 PMC3671721

[28] Dorn-In S, Körner T, Büttner M, Hafner-Marx A, Müller M, Heurich M, et al. Shedding of *Mycobacterium caprae* by wild red deer (*Cervus elaphus*) in the Bavarian alpine regions, Germany. Transbound Emerg Dis. 2020;67:308–17. 10.1111/tbed.13353 31512795

[29] Ghielmetti G, Scherrer S, Friedel U, Frei D, Suter D, Perler L, et al. Epidemiological tracing of bovine tuberculosis in Switzerland, multilocus variable number of tandem repeat analysis of *Mycobacterium bovis* and *Mycobacterium caprae.* PLoS One. 2017;12:e0172474. 10.1371/journal.pone.0172474 28222182 PMC5319696

[30] Fink M, Schleicher C, Gonano M, Prodinger WM, Pacciarini M, Glawischnig W, et al. Red deer as maintenance host for bovine tuberculosis, Alpine region. Emerg Infect Dis. 2015;21:464–7. 10.3201/eid2103.141119 25695273 PMC4344270

[31] Schöning JM, Cerny N, Prohaska S, Wittenbrink MM, Smith NH, Bloemberg G, et al. Surveillance of bovine tuberculosis and risk estimation of a future reservoir formation in wildlife in Switzerland and Liechtenstein. PLoS One. 2013;8:e54253. 10.1371/journal.pone.0054253 23349839 PMC3549981

[32] Leth C, Varadharajan A, Mester P, Fischaleck M, Rossmanith P, Schmoll F, et al. Matrixlysis, an improved sample preparation method for recovery of *Mycobacteria* from animal tissue material. PLoS One. 2017;12:e0181157. 10.1371/journal.pone.0181157 28723969 PMC5517009

[33] Fite GL, Cambre PJ, Turner MH. Procedure for demonstrating lepra bacilli in paraffin sections. Arch Pathol (Chic). 1947;43:624–5. 20252709

[34] Supply P, Allix C, Lesjean S, Cardoso-Oelemann M, Rüsch-Gerdes S, Willery E, et al. Proposal for standardization of optimized mycobacterial interspersed repetitive unit-variable-number tandem repeat typing of *Mycobacterium tuberculosis.* J Clin Microbiol. 2006;44:4498–510. 10.1128/JCM.01392-06 17005759 PMC1698431

[35] Allix-Béguec C, Harmsen D, Weniger T, Supply P, Niemann S. Evaluation and strategy for use of MIRU-VNTR*plus*, a multifunctional database for online analysis of genotyping data and phylogenetic identification of *Mycobacterium tuberculosis* complex isolates. J Clin Microbiol. 2008;46:2692–9. 10.1128/JCM.00540-08 18550737 PMC2519508

[36] Brudey K, Driscoll JR, Rigouts L, Prodinger WM, Gori A, Al-Hajoj SA, et al. *Mycobacterium tuberculosis* complex genetic diversity: mining the fourth international spoligotyping database (SpolDB4) for classification, population genetics and epidemiology. BMC Microbiol. 2006;6:23. 10.1186/1471-2180-6-23 16519816 PMC1468417

[37] Chiari M, Zanoni M, Alborali LG, Zanardi G, Avisani D, Tagliabue S, et al. Isolation of *Mycobacterium caprae* (Lechtal genotype) from red deer (*Cervus elaphus*) in Italy. J Wildl Dis. 2014;50:330–3. 10.7589/2013-06-135 24499334

[38] Griffin JFT, Mackintosh CG. Tuberculosis in deer: perceptions, problems and progress. Vet J. 2000;160:202–19. 10.1053/tvjl.2000.0514 11061957

[39] Michelet L, Conde C, Branger M, Cochard T, Biet F, Boschiroli ML. Transmission network of deer-borne *Mycobacterium bovis* infection revealed by a WGS approach. Microorganisms. 2019;7:687. 10.3390/microorganisms7120687 31842292 PMC6955793

[40] Javed MT, Aranaz A, de Juan L, Bezos J, Romero B, Alvarez J, et al. Improvement of spoligotyping with additional spacer sequences for characterization of *Mycobacterium bovis* and *M. caprae* isolates from Spain. Tuberculosis (Edinb). 2007;87:437–45. 10.1016/j.tube.2007.04.002 17569586

[41] Rodríguez S, Romero B, Bezos J, de Juan L, Alvarez J, Castellanos E, et al.; Spanish Network on Surveillance and Monitoring of Animal Tuberculosis. High spoligotype diversity within a *Mycobacterium bovis* population: clues to understanding the demography of the pathogen in Europe. Vet Microbiol. 2010;141:89–95. 10.1016/j.vetmic.2009.08.007 19720476

[42] Michelet L, de Cruz K, Zanella G, Aaziz R, Bulach T, Karoui C, et al. Infection with *Mycobacterium microti* in animals in France. J Clin Microbiol. 2015;53:981–5. 10.1128/JCM.02713-14 25540404 PMC4390619

[43] Boniotti MB, Gaffuri A, Gelmetti D, Tagliabue S, Chiari M, Mangeli A, et al. Detection and molecular characterization of *Mycobacterium microti* isolates in wild boar from northern Italy. J Clin Microbiol. 2014;52:2834–43. 10.1128/JCM.00440-14 24871212 PMC4136184

[44] Hauer A, De Cruz K, Cochard T, Godreuil S, Karoui C, Henault S, et al. Genetic evolution of *Mycobacterium bovis* causing tuberculosis in livestock and wildlife in France since 1978. PLoS One. 2015;10:e0117103. 10.1371/journal.pone.0117103 25658691 PMC4319773

[45] Réveillaud É, Desvaux S, Boschiroli ML, Hars J, Faure É, Fediaevsky A, et al. Infection of wildlife by *Mycobacterium bovis* in France assessment through a national surveillance system, Sylvatub. Front Vet Sci. 2018;5:262. 10.3389/fvets.2018.00262 30430112 PMC6220493

[46] Hauer A, Michelet L, Cochard T, Branger M, Nunez J, Boschiroli ML, et al. Accurate phylogenetic relationships among *Mycobacterium bovis* strains circulating in France based on whole genome sequencing and single nucleotide polymorphism analysis. Front Microbiol. 2019;10:955. 10.3389/fmicb.2019.00955 31130937 PMC6509552

[47] Hart PD, Sutherland I. BCG and vole bacillus vaccines in the prevention of tuberculosis in adolescence and early adult life. BMJ. 1977;2:293–5. 10.1136/bmj.2.6082.293 326347 PMC1630784

[48] Sula L, Radkovský I. Protective effects of *M. microti* vaccine against tuberculosis. J Hyg Epidemiol Microbiol Immunol. 1976;20:1–6. 944209

[49] Wells AQ. Vaccination with the murine type of tubercle bacillus (vole bacillus). Lancet. 1949;2:53–5. 10.1016/S0140-6736(49)91043-0 18133888

[50] Orgeur M, Frigui W, Pawlik A, Clark S, Williams A, Ates LS, et al. Pathogenomic analyses of *Mycobacterium microti,* an ESX-1-deleted member of the *Mycobacterium tuberculosis* complex causing disease in various hosts. Microb Genom. 2021;7:7. 10.1099/mgen.0.000505 33529148 PMC8208694

